# Phenotypic Variability of LGMD 2C/R5 in a Genetically Homogenous Group of Bulgarian Muslim Roma

**DOI:** 10.3390/genes15091144

**Published:** 2024-08-30

**Authors:** Ani Taneva, David Gresham, Velina Guergueltcheva, Teodora Chamova, Veneta Bojinova, Mariana Gospodinova, Maria Katzarova, Radoslav Petkov, Thomas Voit, Lidia Aneva, Ognyan Asenov, Bilyana Georgieva, Violeta Mihaylova, Stoyan Bichev, Tihomir Todorov, Albena Todorova, Luba Kalaydjieva, Ivailo Tournev

**Affiliations:** 1 Department of Neurology, University Hospital “Alexandrovska”, Medical University Sofia, 1431 Sofia, Bulgariaognqn.asenov1992@mail.bg (O.A.); 2 Center for Genomics and Systems Biology, Department of Biology, New York University, New York, NY 10012, USA; dgresham@nyu.edu; 3 Department of Neurology, University Hospital Sofiamed, 1797 Sofia, Bulgaria; 4 Department of Neurology, Sofia University “St. Kliment Ohridski”, 1504 Sofia, Bulgaria; 5 Clinic of Child Neurology, University Hospital of Neurology and Psychiatry “St’ Naum”, 1113 Sofia, Bulgaria; 6 Diagnostic and Consultative Centre, University Hospital St Ivan Rilski, 1000 Sofia, Bulgaria; 7 Department of Orthopedy, USBALO “Prof. B. Boychev”, Medical University Sofia, 1431 Sofia, Bulgaria; m.katzarova@yahoo.com; 8 National Institute for Health and Care Research Great Ormond Street Hospital Biomedical Research Centre, University College London Great Ormond Street Institute of Child Health, London WC1N 1EH, UK; 9 Great Ormond Street Hospital for Children, NHS Foundation Trust, London WC1N 3JH, UK; 10 Clinical Laboratory, Regional Hospital–Blagoevgrad, 2700 Blagoevgrad, Bulgaria; 11 Department of Medical Chemistry and Biochemistry, Medical University of Sofia, 1431 Sofia, Bulgaria; bgeorgieva@medfac.mu-sofia.bg (B.G.);; 12 Neurocenter, Kantonal Hospital Lucerne, 6000 Lucerne, Switzerland; violetamihaylova@gmail.com; 13 National Genetics Laboratory, University Hospital of Obstetrics and Gynecology–“Maichin Dom”, 1431 Sofia, Bulgaria; 14 Genetic Medico-Diagnostic Laboratory Genica, 1612 Sofia, Bulgaria; 15 Centre for Medical Research, Harry Perkins Institute of Medical Research, The University of Western Australia, Perth 6009, Australia; 16 Department of Cognitive Science and Psychology, New Bulgarian University, 1618 Sofia, Bulgaria

**Keywords:** LGMD 2C/R5, gamma-sarcoglycanopathy, Bulgarian Muslim Roma

## Abstract

Sarcoglycanopathies are among the most frequent and severe forms of autosomal recessive forms of limb-girdle muscular dystrophies (LGMDs) with childhood onset. Four subtypes are known: LGMDR3, LGMDR4, LGMDR5 and LGMDR6, which are caused, respectively, by mutations in the *SGCA*, SGCB, *SGCG* and *SGCD* genes. We present the clinical variability of LGMD 2C/R5 among a genetically homogeneous group of 57 patients, belonging to 35 pedigrees. Molecular genetic analysis showed that all 57 patients were homozygous for the C283Y variant. The muscles of the pelvic girdle and the trunk were affected early and were more severely affected, followed by the shoulder girdle. Macroglossia, hypertrophy of the calves, scapular winging and lumbar hyperlordosis were common in the ambulatory phase. A great intra and interfamilial variability in the clinical presentation of LGMD 2C/R5 was observed, despite having the same underlying molecular defect. Females demonstrated a relatively milder clinical course compared to males. Mean creatine phosphokinase (CK) CK levels were 20 times above normal values. Muscle computer tomography (CT) CT or MRIs showed earlier and more severe involvement of the flexor proximal limb muscles in comparison to extensor muscles.

## 1. Introduction

Sarcoglycanopathies are among the most frequent and severe autosomal recessive limb-girdle muscular dystrophies (LGMDs) with childhood onset. Most patients have a severe and rapid course, leading to loss of independent walking ability before age 30–40 years. On average, the earlier the onset, the more rapid the progression, but in some cases the progression is not linear [1]. Four subtypes are known: LGMDR3, LGMDR4, LGMDR5 and LGMDR6, which are caused, respectively, by mutations in the *SGCA*, *SGCB*, *SGCG*, and *SGCD* genes [2]. These result in misfolding of the four corresponding sarcoglycan protein subunits that form the sarcoglycan complex in the sarcolemma of muscle cells. Defects in any of the sarcoglycan subunits prevent the sarcoglycan complex from assembling properly to support normal cell function. Muscle biopsies from LGMD patients have confirmed that these mutations lead to sarcoglycan protein deficiency in the sarcolemma [3], and 187 unique public DNA variants in the *SGCG* gene have been reported to date in the LOVD database [4].

In 1983, Ben Hamida et al. [5] were the first to describe a severe autosomal recessive limb-girdle muscular dystrophy that was exceptionally common in Tunisia, with an estimated prevalence of 7 × 10^−6^. Later, it was found that in three Tunisian families this disease was linked to the pericentromeric region of chromosome 13q [6]. It was characterized by progressive muscle weakness, onset in early childhood and marked inter- and intra-familial variability over the course of the disease [7,8].

Piccolo et al. [9] studied a severe autosomal recessive muscular dystrophy with sarcoglycan deficiency in seven large, unrelated Roma families from various Western European countries (France, Spain and Italy). Genetic linkage analysis showed that the disease was linked to the locus encoding γ-sarcoglycan on chromosome 13q12. The variant p.C283Y (G > A, affecting codon 283), which leads to the replacement of the conserved cysteine with tyrosine at position 283 in the protein, was identified. All patients were homozygous for the variant, while their parents were heterozygous.

In Eastern Europe, Tournev was the first to identify γ-sarcoglycanopathy in 1997 among Turkish Roma in northeastern Bulgaria [10].

Following the description of the familial Roma mutation p.C283Y in the γ-sarcoglycan gene in LGMD 2C/R5 patients in Western Europe, we have reasoned that, similar to other single gene disorders of the Roma, LGMD 2C/R5 should be expected to have a pan-European distribution, resulting from the historical migration routes of this population. This rationale led us to conduct an extensive search for similarly affected individuals of Romani ethnicity in Bulgaria. Both hospital records and extensive field work were used as a source of information, leading to the identification of 57 living patients with the LGMD 2C/R5 phenotype, homozygous for the mutation p.C283Y, and an additional 17 deceased affected siblings. LGMD 2C/R5 thus appears to be the most common myopathy among the Romani minority of Bulgaria, with a prevalence higher than that of Duchenne muscular dystrophy [11].

LGMD 2C/R5 is more restricted and represents the founding and expansion of the Romani migrational categories 20+ generations ago. LGMD2C/R5 was fully confined to the Balkan and Western European Roma and was not present in Vlax Roma. The mean age of the p.C283Y mutation is ~600 (525–775) years [12]. L. Kalaydjieva was the first to genetically verify the disease in Bulgaria and Todorova et al. introduced routine molecular genetic analysis for the presence of the C283Y variant in Bulgaria [13,14].

The aim of our study is to investigate the phenotypic variability of the largest group of genetically homogenous LGMD 2C/R5 patients in Bulgaria, belonging to the group of the Bulgarian Roma. Our findings could be useful for future research in the field of genetic and epigenetic modifiers and therapeutic options.

## 2. Materials and Methods

Fifty-seven patients (29 men and 28 women) from 35 pedigrees were identified. Catamnestic data were also collected for 17 deceased patients with γ-sarcoglycanopathy occurring in the affected families.

All patients underwent standard clinical and neurological examinations including the Walton functional clinical scale, a manual muscle test using the MRC scale.

Written informed consent was obtained from all participants.

The patients were categorized into three groups according to age at loss of ambulation. Loss of the ability to walk independently before age 13 defines the category of severe progression that characterizes the Duchenne-like phenotype; disability after age 16 characterizes the Becker phenotype; and disability between ages 13 and 16 defines the intermediate phenotype [10].

Laboratory tests, including creatine phosphokinase (CK) measurements, were performed on all patients. The values of CK were calculated to be many times above the normal range. Values below 190 are considered normal. Nerve conduction studies (NCS) and electromyography (EMG) were carried out in all patients. They were conducted at the Department of Neurology, University Hospital Aleksandrovska, and in field studies, with a Dantec-Keypoint portable electromyograph (Natus, Copenhagen, Denmark). ECG and echocardiography were performed on 20 patients, and respiratory function assessments were provided for 48 patients. Computer tomography (CT) or magnetic resonance imaging (MRI) of the major muscles were conducted on 12 patients. Computed tomography (CT) was performed on Aqulion 64 (Toshiba—64 slides), and the muscles of the lower limbs were examined with a 3 Tesla MRI machine (Siemens Verio), Siemens Medical Solutions, Malvern, PA, USA. Muscle morphological changes were graded based on the relative proportion of muscle areas with decreased and normal density: mild (hypodense areas smaller than 50% of normally appearing muscle areas), moderate (hypodense areas from 1/2 to 2/3 of normally appearing muscle areas), and severe (muscles are atrophic and more than 2/3 are replaced by low-density tissues). A molecular-genetic analysis for the presence of the variant p.C283Y was performed on all patients. Blood for DNA extraction, with informed consent for genetic testing, was collected from all patients, their parents, siblings, and other relatives. The molecular genetic testing was conducted in the National Genetic Laboratory at the University Hospital “Maichin Dom,” Medical University-Sofia, and the Centre for Human Genetics, Edith Cowan University, Perth. Given the genetic homogeneity of patients with γ-sarcoglycanopathy, most of them have been studied by direct examination of the C283Y mutation: direct amplification of exon 8 of the g-SG gene was performed on dried blood spots from Guthrie cards, followed by single-stranded conformation polymorphism (SSCP) analysis. The presence of the mutation was confirmed by RsaI restriction digestion. Samples with different SSCP migration patterns were sequenced with an ABI PRISM 310 genetic analyzer, Applied Biosystems, Foster City, CA, USA. 

Immunohistochemical studies of muscles were conducted on 7 patients. Immunohistochemical analysis of protein expression was performed in 6 μm cryostat sections of striated muscle. Protein expression in patient muscle was compared to normal protein expression in healthy control muscle. The expression of the following muscle proteins was investigated: caveolin 3, dystrophin 1,2,3, α-dystroglycan, β-dystroglycan, α-sarcoglycan, β-sarcoglycan, γ-sarcoglycan, δ-sarcoglycan, laminin α2 C-terminal, laminin α2 300 kDa, laminin β1, laminin β2, laminin γ1, emerin, lamin A/C, collagen VI, laminin α5 and utrophin (DRP2).

Mathematical and statistical analyses were performed to assess the significance of various disease-related variables and their interactions.

The statistical methods used were tailored to the tasks and the nature of the data:

▪ Descriptive statistics: frequency distribution of quantitative and qualitative characteristics (gender); arithmetical mean, variance and median (total for the whole group and by gender); analysis of mean values—Levene’s test was used, which automatically corrects the influence of the difference in variances (if a statistically significant one is observed) when comparing mean values by groups.
▪ Correlations (Pearson’s linear).

For this purpose, the standard modules from the SPSS-PC+ were used: Descriptive Statistics, Frequencies, Box-plots; Means—*t*-test, ANOVA; Correlation analysis.

An ethnological study was also conducted to identify the groups among which the disease is prevalent.

## 3. Results

### 3.1. Epidemiological Data

**Figure 1:**
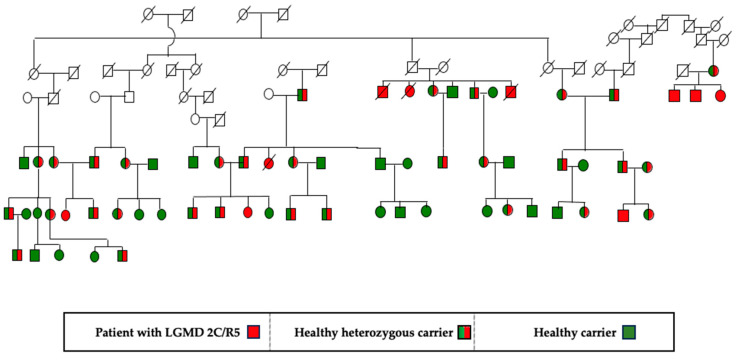
Pedigree with LGMD2C/R5 from the villages of Aprilovo, Golyamo Novo, Elenovo in the Targovishte region, and the villages of Sinya Voda and Rakovski in the Razgrad region.

The clinical cohort included 73 patients with genetically verified γ-sarcoglycanopathy. The age of the patients at the time of the study ranged from 7 to 40 years. Thirty-five pedigrees with the disease were identified. All patients belonged to the group of settled Muslim Roma-Millet. This is a long-settled and long-established group in our lands. Figure 1 presents a pedigree with patients with LGMD 2C/R5.

**Figure 2:**
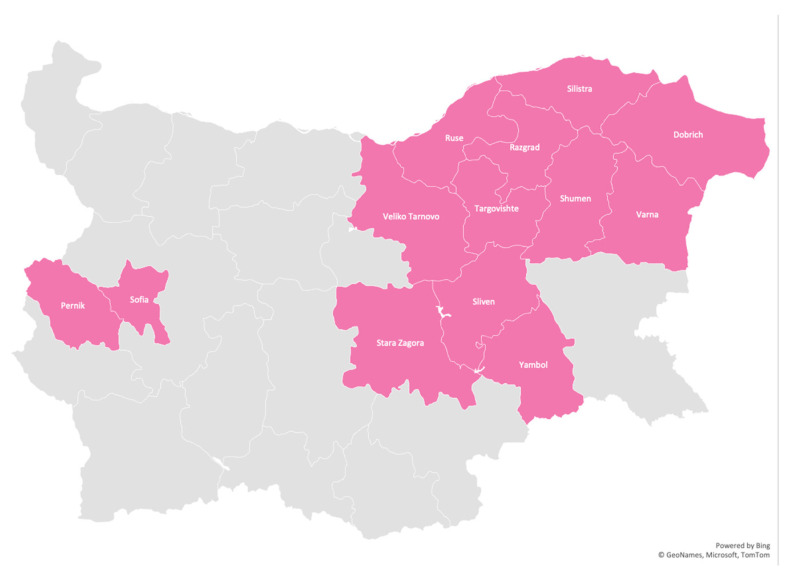
Distribution of LGMD 2C/R5 among Roma in Bulgaria by regions.

The disease has been identified in 13 regions—11 in Eastern Bulgaria: Targovishte, Razgrad, Ruse, Silistra, Dobrich, Varna, Shumen, Veliko Tarnovo, Sliven, Yambol, Stara Zagora, and two in Western Bulgaria: Sofia and Pernik. To a large extent, the distribution of the disease outlines the path of migration. Despite extensive field studies of the disease throughout the country, no cases of LGMD 2C/R5 have been identified in other regions or among other Roma groups. Figure 2 shows the geographic distribution of LGMD 2C/R5 in Bulgaria.

### 3.2. Natural History of the Disease

The disease usually begins with complaints of difficulty walking, frequent falls, walking on tiptoes, difficulty climbing stairs, running, and rising from a squatting position. Summary data on the onset, duration of the disease and average age are presented in Table 1.

Statistically significant sex differences were found during LGMD 2C/R5. The disease starts later in women than in men (*p* < 0.01); men stop walking earlier (*p* < 0.05) than women. The effect of gender on age of disability is also evident from Levene’s test, which shows statistical significance at equal variances—*p* < 0.01. The results of the analysis of variance (ANOVA) also demonstrate statistical significance (*p* < 0.01) of the age at onset X sex and age of disability X sex interactions. Life expectancy was longer in females. There was a statistically significant correlation between age of onset and age of disability (*p* < 0.01). The earlier the onset of the disease, the earlier the disability, i.e., the shorter the duration of walking. The severity of patients’ condition correlated with age at the time of examination (*p* < 0.001).

### 3.3. Clinical and Paraclinical Data

**Figure 3:**
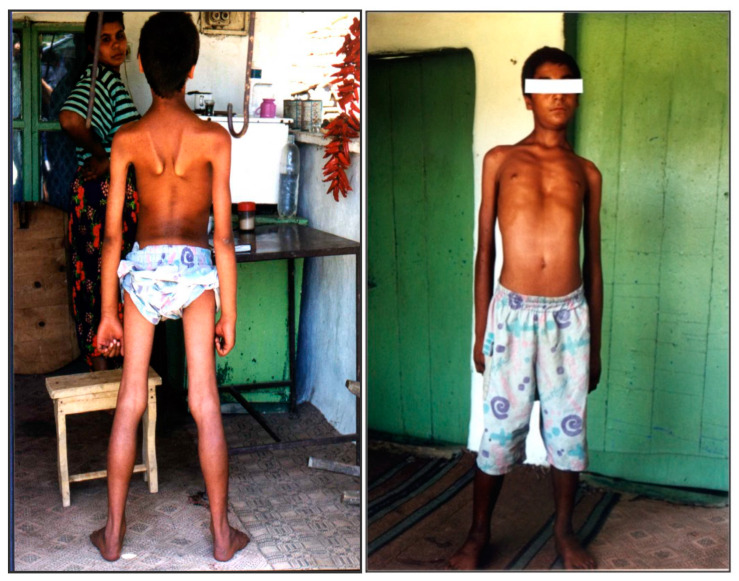
Boy with LGMD 2C/R5 with typical phenotype, 13 years, Maysko village, Elena.

The initial muscle weakness involves the pelvic girdle, followed by the shoulder girdle. At more advanced stages, muscle weakness becomes diffuse, but the mm. glutei, psoas, sacrospinalis, periscapularis and trapezius are the most severely affected. In the proximal limb muscles, there is early selective involvement of the flexors (m. biceps bachii and m. flexor femoris), with relatively good preservation of the extensors (m. triceps brachii and m. quadriceps); Figure 3.

The axial muscles are affected after the proximal muscles, with the occurrence of lumbar hyperlordosis. Calf pseudohypertrophy and macroglossia are typical features of the disease. Macroglossia was found in 21 out of 57 patients (8/28 women and 13/29 men). Distal muscle strength was preserved even in the more advanced stages. No patient was found to have facial or bulbar muscle weakness. Pseudohypertrophy of the hamstrings, scapulae alate and lumbar hyperlordosis were seen in most patients at earlier stages. Progressive scoliosis with significant neck stiffness was found in 18 patients, all of whom were disabled (10 men and 8 women). Ankle joint contractures were found after the age of 8 years in 17/29 males and 15/28 females.

Although there is no difference between the sexes in the type of muscle damage and the degree of its severity, in men, the disease has an earlier onset and more rapid progression. Table 2 shows the percentage of all patients and their gender distribution according to the type of disease.

Additional clinical data are summarized in the following tables Table 2 and Table 3:

EMG was consistent with mixed myogenic and neurogenic changes. Spontaneous activity (fibrillations, positive sharp waves) is found predominantly in mm. biceps brachii, rectus femoris and tibialis anterior. Table 4 shows that the duration of action potentials (AP) is shortened, and the amplitudes of the action potentials were in the region of the lower limit of normal in the automatic analysis. Conduction velocities along motor and sensory fibres were within normal limits; Table 4.

CT and MRI of lower limb muscles were performed in 12 patients. The degree and extent of muscle hypodensity or atrophy varies with the patient’s age and functional ability. There was symmetrical involvement of the lumbar musculature, with changes progressing distally—more distal muscle groups persisting longer in the course of the disease. The mm. glutei (medius and minimus) were minimally affected in the two younger patients, who could independently climb stairs; moderately affected in the 10- to 12-year-old patients who were still walking independently; and severely affected in patients who were unable to walk, being completely replaced by adipose tissue in the oldest and most severely debilitated patients. The m. subscapularis and m. trapezius were minimally affected in younger patients, in whom the m. deltoideus was preserved; severely affected between the ages of 12 and 14, when the m. deltoideus was minimally to moderately affected; and completely atrophic in the oldest patients, in whom only traces of the m. deltoideus were found. Selectively affected muscles in all the patients studied were the mm. glutei, mm. adductori, flexor femoris, abdominalis, spinalis, supraspinatus, infraspinatus, subscapularis and soleus. In the earlier stages, relatively more severe involvement of the flexor than extensor muscle groups were found in all four limbs. At the stage of complete debilitation, most muscles were atrophic or completely replaced by adipose tissue, whereas the mm. deltoideus, quadriceps, sartorius, gracilis and gastrocnemius were relatively preserved.

**Figure 4:**
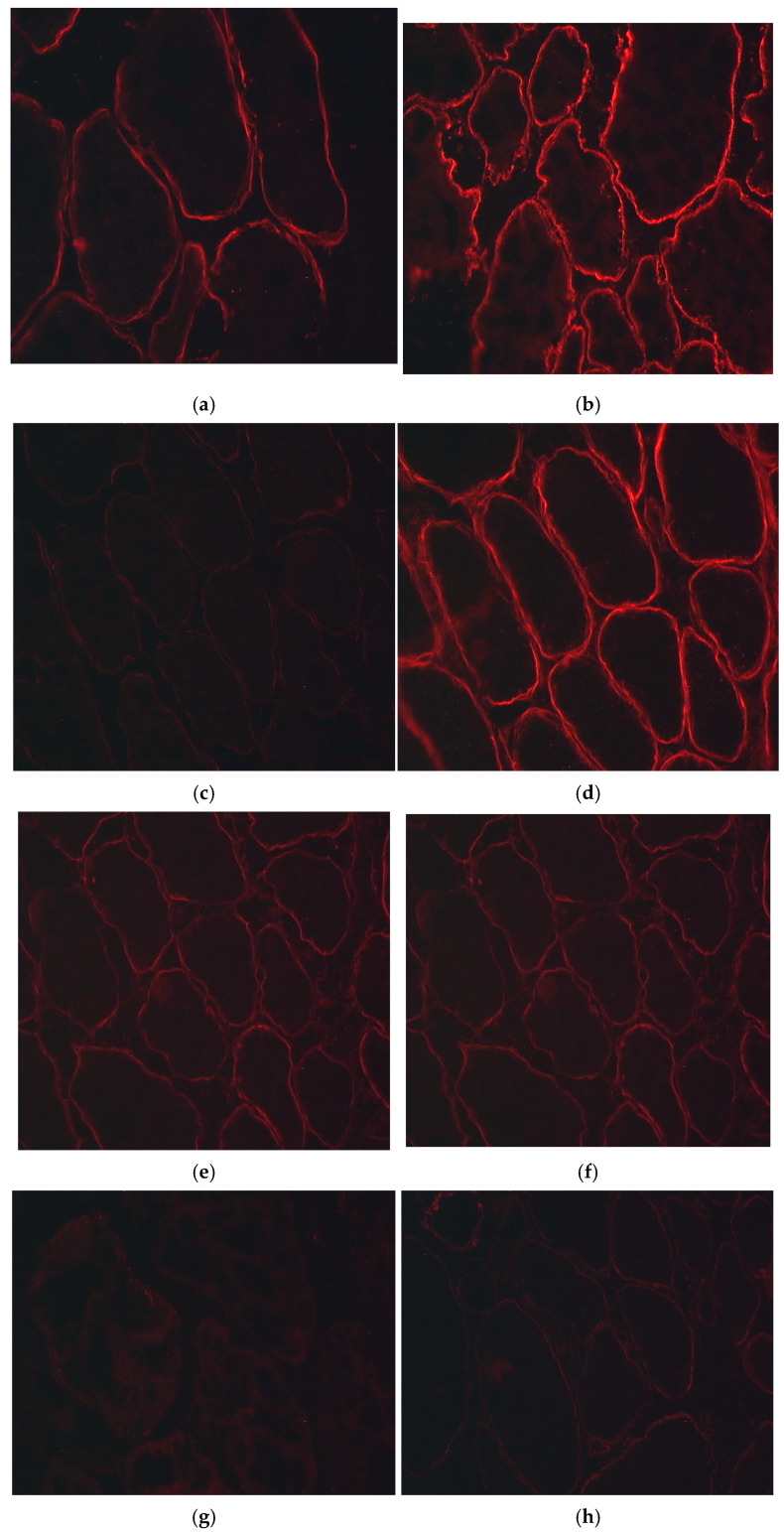
Immunohistochemical analysis of the expression of the following muscle proteins: (**a**) Dystrophin, (**b**) Caveolin, (**c**) α-dystroglycan, (**d**) β-ystroglycan, (**e**) α-sarcoglycan, (**f**) β-sarcoglycan, (**g**) γ-sarcoglycan and (**h**) δ-sarcoglycan.

The immunohistochemical characterization of sarcoglycan and dystroglycan components was studied in seven patients with LGMD 2C/R5. Similar changes were found in all the patients studied: a persistent absence of α-dystroglycan and well-preserved β-dystroglycan. By using different domain-specific antibodies against α-sarcoglycan, we found relative preservation of its intramembrane domain and the absence of its C- and N-terminal parts. β-sarcoglycan was severely reduced or absent. The γ- sarcoglycan was also completely absent in all patients. δ-sarcoglycan was relatively the best preserved, and was expressed to a significantly greater extent in LGMD 2C/R5 than in the Duchenne-like type. These changes can be observed in Figure 4.

Significant inter- and intra-familial variations in the course of the disease were found. In three families with patients of both sexes (1F + 2M; 1F + 1M; 1F + 2M), the disease course was significantly milder and more delayed in women than in men. In a fourth family with LGMD 2C/R5 (1F + 2M), the disease had a more severe course in one brother and sister than in the other brother. In two families in which the patients were only female (2F; 2F), phenotypic variations were also found—in one family the disease had the same onset in both sisters, but with earlier disability in the older sister; in the other family, the course was more severe (with earlier onset and earlier disability) in the younger sister. In two other families in which the patients were only male (4M; 2M), the phenotypic variations were significantly less pronounced.

### 3.4. Genetic Findings

The mutation p.C283Y was found in all patients.

Thirty five pedigrees with the disease were identified. All patients’ parents shared no complaints and had normal neurological status. Two parental pairs were also tested for CK values, which were normal. LGMD 2C/R5 has an autosomal recessive type of inheritance. A detailed pedigree analysis was performed in each family; one is presented in Figure 1.

## 4. Discussion

LGMD 2C/R5 is the most common myopathy among Roma in Bulgaria, although it has a regional prevalence in the eastern part of the country. All patients belong to the group of settled Muslim Roma-Millet. No cases of LGMD 2C/R5 have been identified in other Roma groups. The study of affected families included a genetically homogeneous group of 57 patients with LGMD 2C/R5, all confirmed homozygous for the C283Y mutation. The disease has been identified in only 13 regions, 11 of them of Eastern Bulgaria.

Pseudohypertrophy of the lower limbs, scapular winging, lumbar hyperlordosis and maroglossia were typically observed in the earlier stages. Clinical examination and muscle imaging show early selective involvement of the mm. glutei, adductors, flexor femoris, abdominalis, spinalis, subscapularis and soleus, and relative sparing of the m. quadriceps. Flexor muscle groups are more severely affected than extensor muscles. Our clinical observations in LGMD 2C/R5 patients are similar to the findings of a joint European study [15] regarding the natural history of the disease, the rate of progression of the disability, the pattern of muscle involvement and the absence of cognitive impairment and cardiomyopathy. Restrictive-type respiratory failure with significant respiratory events usually is a common finding [16,17], but only one patient with severe respiratory failure requiring invasive pulmonary ventilation was found in the Bulgarian population. The described Duchenne-like phenotype was seen in the largest percentage of patients, with the remainder distributed almost equally between those classified with moderate disease severity and those with a milder phenotype similar to Becker-type MD, despite all patients having the same mutation in the homozygous state.

Our findings in the genetically homogeneous group of LGMD2C/R5 Roma patients, all homozygous for the C283Y mutation, add further evidence of the existence of factors modifying disease severity. Differences were observed in some of our affected pedigrees—two families presented with all three phenotypes in three affected children in each family and five additional families exhibited manifestations of two phenotypes in them. These differences may be related to the degree of involvement of other sarcoglycan components or may be due to the influence of modifying genes or environmental factors. Importantly, our study confirms the claim of Alonso-Perez et al. on the significance of symptom onset before 10 years of age as an independent risk factor for loss of mobility before 18 years of age in patients with LGMD 2C/R5 [18].

It can be assumed that the C283Y mutation leads to a unified type of immunohistochemical changes, expressed in the loss of α-dystroglycan and β- and γ-sarcoglycan, with relative preservation of α- and δ-sarcoglycan. Dystrophin deficiency in Duchenne-like type PMD conversely results in a predominant loss of α- and δ-sarcoglycans, but also the loss of α-dystroglycan. However, in γ-sarcoglycan-deficient patients, trace amounts of residual sarcoglycans are evident at the cell membrane, whereas no consequences for the DGC or a reduction of dystrophin are reported [19].

Males were found to have an earlier onset and more rapid progression. Although no sex-related differences were observed with respect to muscle involvement, loss of independent gait occurred earlier in men. Milder phenotypes were significantly more common among female patients, with the exception of one representative, in whom severe respiratory failure with the need for IVF occurred. Gender differences with more severe male involvement were also reported by G. Leal and E. Da Silva [20] in a Brazilian family with γ-sarcoglycanopathy caused by the del521T variant. The authors found sex differences in only one of the four families studied. It was noted in the collaborative Merlini et al. study [21], which however failed to show statistically significant sex-related differences. Given the increasing awareness of the existence of genetic factors modifying disease progression, such findings are an important indication that the genes involved may be X-linked or under hormonal influence [10].

Ben Hamida et al. [5] described significant inter- and intra-familial variation, but found no sex differences. Such variations were also observed in the families we studied, even among patients of the same sex.

In LGMD 2C/R5, compared to Duchenne muscular dystrophy, scoliosis develops later and to a lesser degree. Due to this fact, respiratory complications occur most often after the age of 30. It is known that life expectancy in Duchenne MD is severely limited, and that only a minority of patients survive more than 25 years without mechanical ventilation [22]. In γ-sarcoglycanopathy with the variant p.C283Y, 25% of patients survive to this age. The higher life expectancy is most likely due to the absence of early respiratory disorders and heart failure.

γ-sarcoglycanopathy with the pathogenic variant p.C283Y has a more severe course than that caused by the del521T variant seen in North Africa [4]. Only 20% of Tunisian patients lose the ability to walk independently by the age of 15 years, compared with 81.5% of patients with the “private“ Roma mutation. The phenotype in Roma and Tunisians is similar, except that the macroglossia is not found in the latter and cardiomyopathy is more commonly reported.

## 5. Conclusions

LGMD 2C/R5 in Bulgaria is described in a large group of settled Muslim Roma-Millets, inhabiting mainly the eastern part of the country. All are carriers of the same pathogenic variant p.C283Y in a homozygous state, described as a “private” Roma mutation. The clinical phenotype follows the natural history of the disease, with a great phenotypic variability observed both intra- and inter-familiarly, despite the same underlying molecular defect. Statistically significant sex differences in the course of the disease were found. The disease is more severe in men than in women. Although there is no difference between the sexes in the type of muscle damage and the degree of severity, the disease has an earlier onset in men and leads to more rapid disability. Females demonstrated a relatively milder clinical course of the disease. Further studies in a larger number of LGMD2C patients are needed to fully describe the phenotypic spectrum of this and other mutations in the SGCG gene. Research on epigenetic factors relevant to sex differences is also needed for a more complete assessment. This can be useful for future research in the field of genetic and epigenetic modifiers and therapeutic options.

## Figures and Tables

**Table 1:** Distribution by sex, age of onset, duration of disease, gait loss and death of the cohort of patients with LGMD 2C/R5.

	All Patients	Female	Male
Mean age at the time of the study	21.9 ± 7.8	22.9 ± 8.7	20.9 ± 7.0
Mean age of onset of the disease (between 2 and 13 yrs of age)	6.7 ± 2.5	7.8 ± 2.5	5.7 ± 2.0
Mean age of lost ambulation	13.6 ± 3.2	14.9 ± 3.7	12.4 ± 2.1
Average duration of ability to walk after disease onset	6.9 ± 3.1	7.1. ± 3.8	6.7. ± 2.3
Mean age of death	28.13 ± 3.5	32.6 ± 3.1	25.9 ± 3.8

**Table 2:** Percentage of patients in relation to the clinical course of LGMD 2C/R5.

	% All Patients	% Female	% Male
Duchenne-like phenotype	52.5	36,8	66.7
Intermediate phenotype	27.5	26.3	28.6
Becker-like phenotype	20	36.8	4.8

**Table 3:** Summarized Clinical Data.

	Number of Patients Studied	Results			Additional Characteristics:
CK values	All patients	Mean value of CK is 20 times the norm			Normal range <190 U/I
Intellectual functions	All patients	Preserved in all patients			
Cardiac functions	20 patients	One with dilated cardiomyopathy and marked systolic dysfunction requiring medical therapy			Patients studied are aged 10–40 yrs of age
Respiratory functions	48 patients	Six with mild restrictive type of respiratory disorder	One with severe respiratory failure	One with invasive pulmonary ventilation treatment from 35 yrs of age	Mean value of FVC is 70%.

**Table 4:** Summarized EMG data.

	m. Rectus Femoris	m. Tibialis Anterior	m. Triceps Brachii	m. Biceps Brachii
Duration of AP	2.0–3.8 ms	2.5–3.3 ms	4.5–8.6 ms	2.0–3.3 ms
Amplitudes of the AP	<0.9 mV/0.7–0.9/	<0.7 mV/0.3–0.7/	<0.9 mV/0.7–0.9/	<0.4 mV/0.2–0.4/

## Data Availability

The original contributions presented in the study are included in the article, further inquiries can be directed to the corresponding author.
